# Efficient Inhibition of HIV Replication in the Gastrointestinal and Female Reproductive Tracts of Humanized BLT Mice by EFdA

**DOI:** 10.1371/journal.pone.0159517

**Published:** 2016-07-20

**Authors:** Uma Shanmugasundaram, Martina Kovarova, Phong T. Ho, Nathaniel Schramm, Angela Wahl, Michael A. Parniak, J. Victor Garcia

**Affiliations:** 1 Division of Infectious Diseases, Center for AIDS Research, University of North Carolina at Chapel Hill, School of Medicine, Chapel Hill, North Carolina, United States of America; 2 Department of Microbiology and Molecular Genetics, University of Pittsburgh School of Medicine, Pittsburgh, Pennsylvania, United States of America; Burnet Institute, AUSTRALIA

## Abstract

**Background:**

The nucleoside reverse transcriptase inhibitor (NRTI) 4'-ethynyl-2-fluoro-2'-deoxyadenosine (EFdA) in preclinical development exhibits improved safety and antiviral activity profiles with minimal drug resistance compared to approved NRTIs. However, the systemic antiviral efficacy of EFdA has not been fully evaluated. In this study, we utilized bone marrow/liver/thymus (BLT) humanized mice to investigate the systemic effect of EFdA treatment on HIV replication and CD4+ T cell depletion in the peripheral blood (PB) and tissues. In particular, we performed a comprehensive analysis of the female reproductive tract (FRT) and gastrointestinal (GI) tract, major sites of transmission, viral replication, and CD4+ T cell depletion and where some current antiretroviral drugs have a sub-optimal effect.

**Results:**

EFdA treatment resulted in reduction of HIV-RNA in PB to undetectable levels in the majority of treated mice by 3 weeks post-treatment. HIV-RNA levels in cervicovaginal lavage of EFdA-treated BLT mice also declined to undetectable levels demonstrating strong penetration of EFdA into the FRT. Our results also demonstrate a strong systemic suppression of HIV replication in all tissues analyzed. In particular, we observed more than a 2-log difference in HIV-RNA levels in the GI tract and FRT of EFdA-treated BLT mice compared to untreated HIV-infected control mice. In addition, HIV-RNA was also significantly lower in the lymph nodes, liver, lung, spleen of EFdA-treated BLT mice compared to untreated HIV-infected control mice. Furthermore, EFdA treatment prevented the depletion of CD4+ T cells in the PB, mucosal tissues and lymphoid tissues.

**Conclusion:**

Our findings indicate that EFdA is highly effective in controlling viral replication and preserving CD4+ T cells in particular with high efficiency in the GI and FRT tract. Thus, EFdA represents a strong potential candidate for further development as a part of antiretroviral therapy regimens.

## Introduction

Current antiretroviral therapy (ART) regimens effectively control peripheral blood (PB) plasma viral load levels and decrease morbidity and mortality in HIV-infected patients. However, due to limited penetration of ART, HIV replication can persist in tissue reservoirs such as the gastrointestinal (GI) tract and female reproductive tract (FRT) and lymphoid tissues [1–3]. Anti-HIV drugs with poor tissue penetrance may also contribute to the development of drug resistant variants, inflammation, maintenance of viral reservoirs, and HIV transmission [4, 5]. Therefore, new drugs with strong penetration into these tissues are crucial for more effective HIV treatment, prevention, and eradication/cure strategies.

The nucleoside reverse transcriptase inhibitor (NRTI) 4'-ethynyl-2-fluoro-2'- deoxyadenosine (EFdA), currently in preclinical development, has potent antiviral activity with improved safety and minimal drug resistance compared to other approved NRTIs [6]. In *vitro* efficacy studies have demonstrated that EFdA inhibits HIV-1 replication in primary peripheral blood mononuclear cells (PBMC) at a 50% effective concentration (EC50) of 50 pM, a potency 4-fold greater than Tenofovir (TFV) and 400-fold greater than azidothymidine (AZT) [7]. EFdA is non-toxic *in vitro* at concentrations as high as 10 μM, with a selectivity index greater than 200,000 [6, 8]. EFdA exhibited increased potency in blocking simian immunodeficiency virus (SIV) replication *in vitro* in primary macaque PBMC compared to TFV, AZT and emtricitabine (FTC). It also showed efficient inhibition of replication *in vivo* in two SIV-infected macaques with advanced acquired immunodeficiency syndrome (AIDS) [9]. In HIV-infected NOD/SCID Janus kinase 3 knockout mice injected with human PBMC, EFdA treatment reduced plasma HIV-RNA levels and prevented CD4+ T cell depletion in PB [10]. In addition, a recent study demonstrated that EFdA decreased HIV replication *in vitro* in human primary lymphocytes infected with multiple clade HIV isolates and in plasma of humanized mice infected with an early passage HIV isolate [11].

Despite these studies, the effect of EFdA on systemic HIV replication, specifically in highly relevant mucosal tissues where transmission can occur, has not been documented. In the present study, we used bone marrow/liver/thymus (BLT) [12–16] humanized mice to analyze the anti-HIV activity of EFdA in tissues with particular emphasis on the FRT and GI tract. We administered EFdA (10mg/kg) orally to HIV-infected BLT mice once daily and monitored HIV-RNA levels in plasma and cervicovaginal lavage (CVL). Following three weeks of EFdA therapy, HIV-RNA and HIV-DNA in plasma, CVL and multiple tissues including the GI tract and FRT demonstrated a significantly lower compared to untreated controls. Our findings indicate that EFdA is a promising antiviral candidate for HIV treatment and prevention strategies.

## Materials and Methods

### Generation of BLT humanized mice

BLT mice were prepared as previously described [17, 18]. Briefly, thymus/liver/thymus implanted NOD/SCIDγ_c_^-/-^ (NSG; The Jackson Laboratories) were transplanted with autologous human liver-derived CD34+ hematopoietic stem cells (Advanced Bioscience Resources, Alameda, CA) and reconstitution of human immune cells in PB was analyzed by flow cytometry, as we previously described [19–21]. Mice were maintained under specific-pathogen-free conditions by the Division of Laboratory Animal Medicine according to protocols approved by the Institutional Animal Care and Use Committee at the University of North Carolina–Chapel Hill.

### Virus challenge and administration of EFdA

Stocks of HIV-1_JR-CSF_ were prepared via transient transfection of 293 T cells, and titred using TZM-bl cells as previously described [22]. HIV-1_JR-CSF_ (30,000 TCIU) was administered intravenously by tail vein injection.

EFdA was generously provided by Michael A. Parniak, University of Pittsburgh School of Medicine. EFdA was reconstituted in phosphate-buffered saline (PBS) at a concentration of 1 mg/mL and administered orally to BLT mice by oral gavage at 10 mg/kg once daily for 3 weeks beginning at 3 weeks post-HIV infection. PBS (200 μL) was administered by oral gavage to (untreated) controls.

### Specimen collection and processing

PB and CVL samples were collected longitudinally (weekly) pre- and post-HIV exposure for 6 weeks. PB was collected in EDTA and plasma separated for HIV-RNA analysis by centrifuging for 5 min at 300 g. The remaining blood cells were reconstituted with PBS to restore the original volume of the PB sample and used for flow cytometric analysis. Cervicovaginal secretions (CVS) were obtained by performing a cervicovaginal lavage (CVL, weeks 0–5) with sterile PBS (3 washes of 20 μl each, ~ 60 μl total volume). To ensure that the procedure was atraumatic, CVL were performed with 20 μl sterile filter pipette tips that were inserted no more than 1–3 mm into the vaginal cavity. Following centrifugation (300g for 5 min), cell-free supernatants were used for HIV-RNA analysis. Pellets were re-suspended in PBS and used for flow cytometric analyses. The bone marrow (BM), LN, human thymic organoid (ORG), liver, lung, spleen, GI tract (from duodenum to rectum) and FRT (vagina, cervix and uterus) were harvested at necropsy 6 weeks post-HIV exposure and mononuclear cells were isolated as previously described [19, 21, 23] for HIV-RNA, HIV-DNA and flow cytometric analyses.

### HIV viral load and flow cytometry analysis

PB and CVL HIV-RNA levels were measured using one-step reverse transcriptase real-time PCR [ABI custom TaqMan Assays-by-Design (limit of detection (LOD): plasma-750 copies/ml, CVL-1400 copies/60μl) [24, 25]. Plasma and CVL viral load levels below the limit of detection were plotted as 375 copies/ml and 700 copies/ml respectively. We used these values to calculate means for the groups. The presence of HIV-RNA and HIV-DNA in mononuclear cells isolated from tissues were determined by real-time RT-PCR (HIV-RNA, LOD-1.5 copies/10^5^cells and HIV-DNA, LOD of 2.5 copies/10^5^cells) [19, 20]. As a control for the presence of DNA extracted from human cells, all samples were tested for the presence of human gamma globin DNA by real-time PCR.

Percentage of CD4+ human T cells were determined by flow cytometry using the following antibodies directed against human CD4 PerCP (SK3), CD3 PE-Cy7 (SK7), and CD45 APC-Cy7 (2D1) (BD Biosciences). Data was acquired with a BD FACSCanto flow cytometer and analyzed with BD FACS Diva software (v. 6.1.3) as described previously [26]. Flow cytometric gating for CD4+ T cells was performed first by gating the live cells as determined by forward and side scatter and then on human CD45+ CD3+ T cells. The mean CD45+ events collected during flow cytometry were 12511 (1380–23881) for PB, 41938 (17058–54998) for ORG, 25733 (2800–50526) for spleen, 27060 (12103–39353) for lung, 17332 (3628–42965) for LN, 58933 (14323–8830) for liver, 21234 (12571–47231) for BM, 723 (368–1851) for GI and 3229 (1473–6202) for FRT. The mean CD3+ events were 10371 (980–21106) for PB, 39725 (17000–51946) for ORG, 19780 (1439–34872) for spleen, 15439 (4724–35362) for lung, 17638 (2639–39823) for LN, 42463 (8349–65526) for liver, 3796 (1474–10294) for BM, 591 (213–1335) for GI, 1358 (509–3758) for FRT.

### Statistical analysis

Repeated measures of two-way ANOVA was used to compare the levels of HIV-RNA in plasma and CVL between EFdA-treated and untreated mice. A Mann-Whitney test was used to compare the mean percentage of CD4+ T cells in the PB and CVL, and to compare HIV-RNA and HIV-DNA levels in the tissues of EFdA-treated and untreated HIV-infected BLT mice. Wilcoxon matched-pairs signed rank test was used to compare CD4+ T cell levels in the PB and CVL of BLT mice pre- and post HIV infection. All data is plotted as mean ± S.E.M and p-values <0.05 were considered significant. All statistical tests were performed using GraphPad Prism software v6.

## Results

### Experimental Design

**Fig 1:**
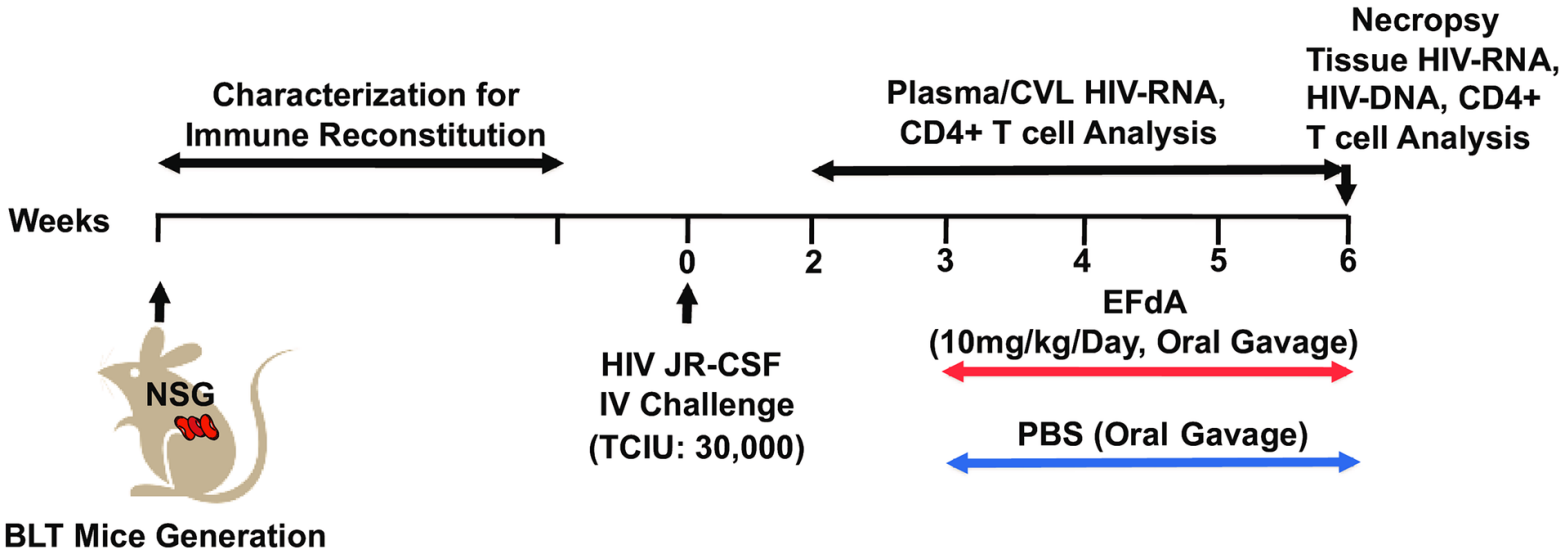
Experimental Design. NSG mice were used to construct BLT humanized mice. The peripheral blood (PB) of BLT mice were monitored longitudinally for human immune reconstitution. BLT mice were infected with HIV-1_JR-CSF_ (30,000 TCIU) intravenously (IV, day 0). Beginning at 3 weeks post-HIV infection, BLT mice (n = 6) were administered EFdA (10mg/kg) once daily for three weeks via oral gavage. Control (untreated) mice (n = 6) were administered PBS orally. PB and CVL were collected weekly from EFdA-treated and untreated mice for the analysis of HIV-RNA and CD4+ T cell levels. EFdA-treated and untreated mice were necropsied at 6 weeks post-infection and tissues harvested for analysis of HIV-RNA, HIV-DNA and CD4+ T cell levels.

The detailed experimental approach for the experiments described below is depicted in Fig 1. Humanized BLT mice were constructed and characterized as previously described [19–21]. Twelve BLT mice were infected with HIV-1_JR-CSF_ (30,000 TCIU) intravenously at week 0. Six BLT mice were included in the EFdA-treated group and 6 mice served as untreated controls. Beginning at three weeks post-infection, the treatment group was administered EFdA (10mg/kg) once daily by oral gavage for 3 weeks. PBS was administered by oral gavage to controls (untreated). PB and CVL were collected from EFdA-treated and untreated mice weekly to measure viral load levels. Multiple organs (BM, LN, ORG, liver, lung, spleen, GI tract and FRT) were collected from EFdA-treated and untreated BLT mice at necropsy, 6 weeks post-infection (3 weeks following EFdA treatment initiation) to quantitate the levels of HIV-RNA, HIV-DNA, and CD4+ T cells.

The BLT mice (n = 12) used for these experiments were characterized for human immune reconstitution in PB prior to HIV infection. Mice were well reconstituted with human hematopoietic cells (CD45+, median 73.3%, range 34–91%). The majority of human hematopoietic cells in PB were human T cells (68.2% range 39–80%). Of the human T cells present, 79.5% (range 65–95%) were CD4+ T cells (Table 1).

**Table 1:** Humanization and HIV-RNA Levels in HIV-infected BLT Mice.

BLT Mouse No.	Study Group	Peripheral blood humanization			HIV-RNA levels	
		%hCD45	%hCD3	%hCD4	Plasma copies/ml (week 6)	CVL copies/60μl (week 5)
1	Treated	66.1	80	82	1074	<LOD
2	Treated	67.1	73.6	82.3	1297	<LOD
3	Treated	69.2	70.8	80.4	<LOD	<LOD
4	Treated	76.6	68.4	80.4	<LOD	<LOD
5	Treated	81.2	62.8	82.2	<LOD	<LOD
6	Treated	63.9	38.5	76.1	<LOD	<LOD
7	Control	77.6	71.8	78.9	6.3x10^5^	1.4x10^5^
8	Control	86.2	43.1	74.7	14.5x10^5^	0.18x10^5^
9	Control	76.7	68	83.9	1.3x10^5^	3086
10	Control	91.3	67.3	65.4	58x10^5^	9576
11	Control	70.1	39.8	73.1	18x10^5^	9667
12	Control	33.6	77.5	95	17x10^5^	18461

Notes: hCD45- Human CD45, hCD3-Human CD3, hCD4-Human CD4, LOD- limit of detection.

### EFdA therapy efficiently reduces plasma HIV-RNA levels

**Fig 2:**
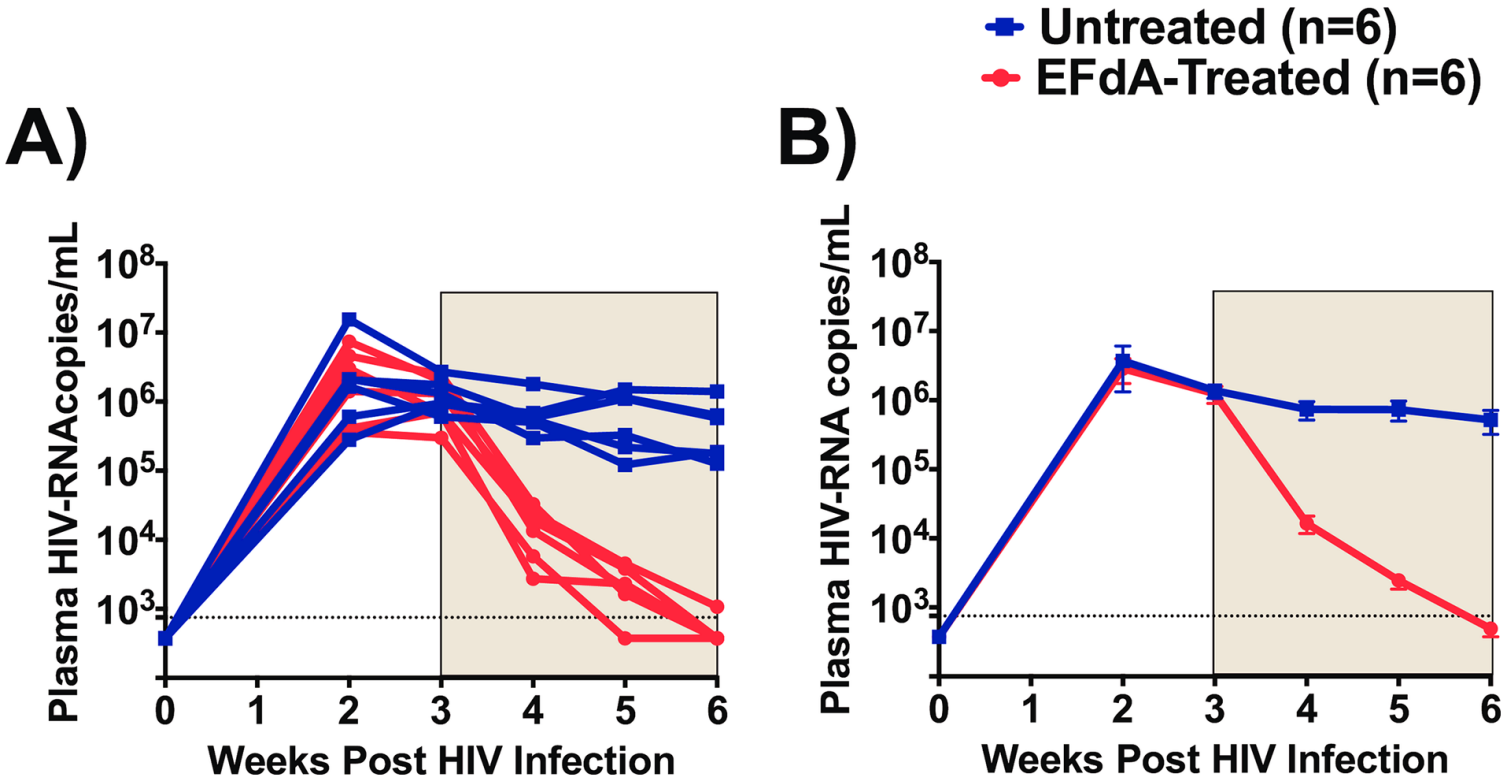
Effect of EFdA administration on the levels of plasma HIV-RNA. A) HIV-RNA levels in the plasma of EFdA-treated (n = 6, red lines) and untreated (n = 6, blue lines) mice. B) Mean plasma HIV-RNA levels in EFdA-treated (red line) and untreated (blue line) mice from Panel A. Shaded area indicates the weeks of EFdA (treated) and PBS (untreated controls) administration. Repeated measures of two-way ANOVA was used to compare the mean levels of HIV-RNA between EFdA-treated and untreated mice. Horizontal and vertical lines indicate mean and standard error. Dotted lines indicate the limit of detection (750 copies/ml).

To determine the systemic antiviral effects of EFdA, we longitudinally monitored HIV-RNA levels in the plasma of EFdA-treated and untreated (Fig 2). At the time of treatment initiation (week 3 post-infection), mean plasma HIV-RNA levels in the EFdA treatment and control groups of mice were similar (p = 0.71). Specifically, the mean viral load for mice in the EFdA treatment and control groups were 1.2 x10^6^ (range 0.3–2.4x10^6^) and 1.4 x10^6^ (range 0.6–2.6x10^6^) copies per ml plasma respectively. After one week of EFdA treatment, we observed a dramatic 2-log reduction (mean 1.6x10^4^, range 0.2–3.2x10^4^ copies/ml, p<0.0001) in plasma HIV-RNA levels in EFdA-treated mice (Fig 2B). No reduction in plasma HIV-RNA levels was noted in untreated control mice. After three weeks of EFdA treatment, the plasma HIV-RNA levels in four EFdA-treated mice decreased below LOD. The plasma HIV-RNA levels in the two EFdA-treated mice with detectable HIV-RNA were reduced by 3-logs to 1074 and 1297 copies/ml after three weeks of EFdA treatment (Fig 2A).

### EFdA effectively reduces HIV-RNA and HIV-DNA levels in lymphoid tissues and effector sites

**Fig 3:**
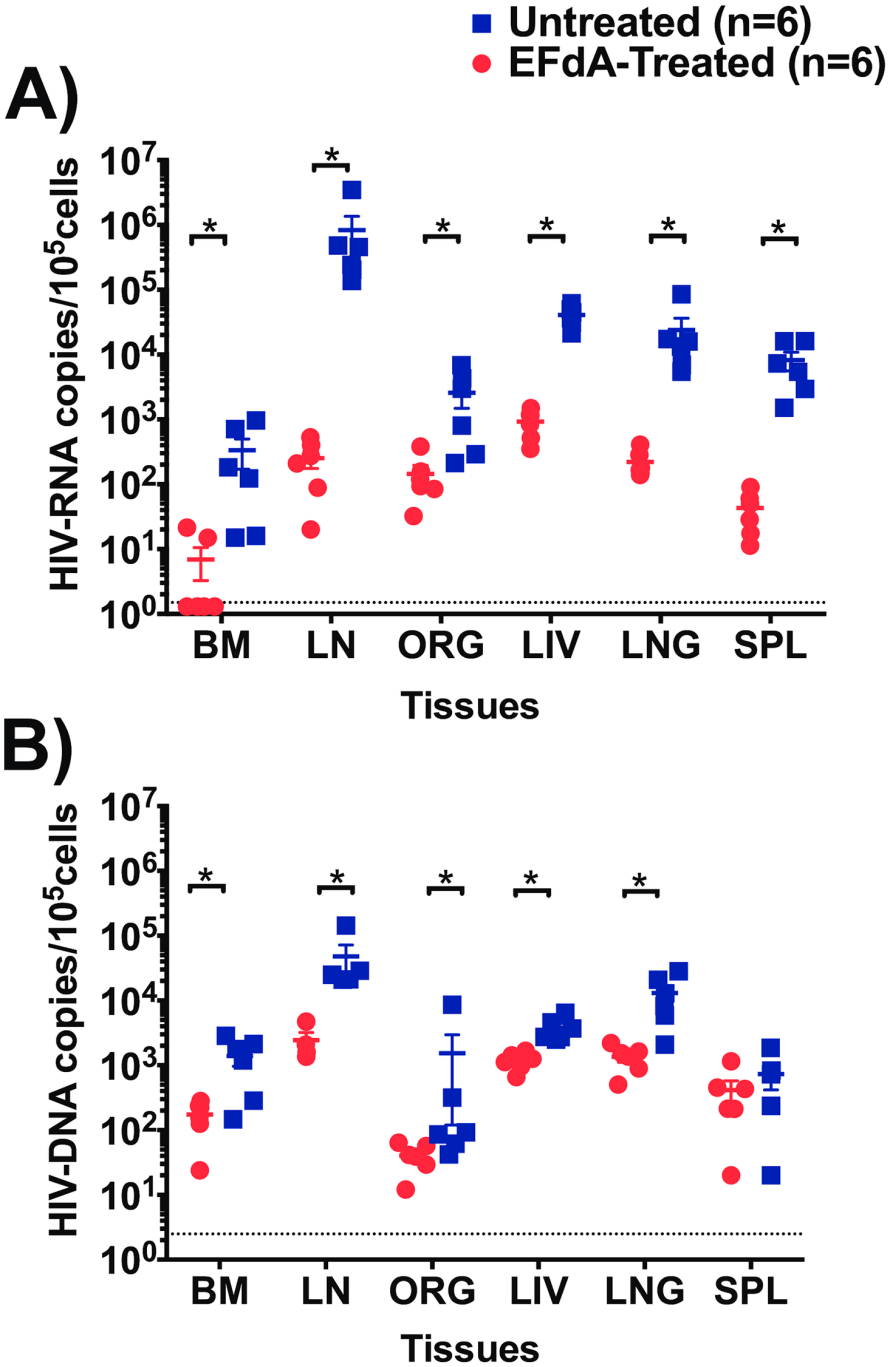
Analysis of HIV-RNA and HIV-DNA levels in lymphoid and effector tissues of EFdA-treated and untreated HIV-infected BLT mice. A) HIV-RNA and B) HIV-DNA levels in the bone marrow (BM), lymph node (LN), human thymic organoid (ORG), liver, lung, spleen, of EFdA-treated (n = 6, circles, red) and untreated (n = 6, square, blue) HIV-infected BLT mice. Dotted lines indicate the limit of detection (HIV-RNA: 1.5 copies/10^5^ cells, HIV-DNA: 2.5 copies/10^5^ cells). Horizontal and vertical lines indicate mean and standard error. A Mann-Whitney test was used to compare HIV-RNA and HIV-DNA levels between EFdA-treated and untreated BLT mice (*p<0.05).

Several studies have shown high levels of HIV-RNA and HIV-DNA in tissues such as LN, spleen, liver and lung despite suppressive ART [2–4, 27, 28]. We therefore determined the systemic effect of EFdA on HIV-RNA and HIV-DNA levels in primary and secondary lymphoid tissues and in effector sites, by comparing the quantities of cell-associated HIV-RNA and HIV-DNA in tissues of EFdA-treated and untreated HIV-infected BLT mice (Fig 3). Cell-associated HIV-RNA levels in the tissues of EFdA-treated BLT mice were significantly lower compared to untreated mice (p = 0.01 for BM, p = 0.002 for ORG, LN, liver, lung, spleen). Notably, we observed a 3-log difference in the levels of HIV-RNA in the LN of EFdA-treated mice compared to untreated mice (Fig 3A). EFdA treatment also resulted in significantly lower HIV-DNA levels in the BM (p = 0.02), LN (p = 0.01), liver (p = 0.002), and lung (p = 0.004) of EFdA-treated mice compared to untreated mice (Fig 3B). Together, these data indicate that EFdA effectively controls HIV replication systemically.

### CD4+ T cell levels in the PB, lymphoid and effector tissues during EFdA treatment

**Fig 4:**
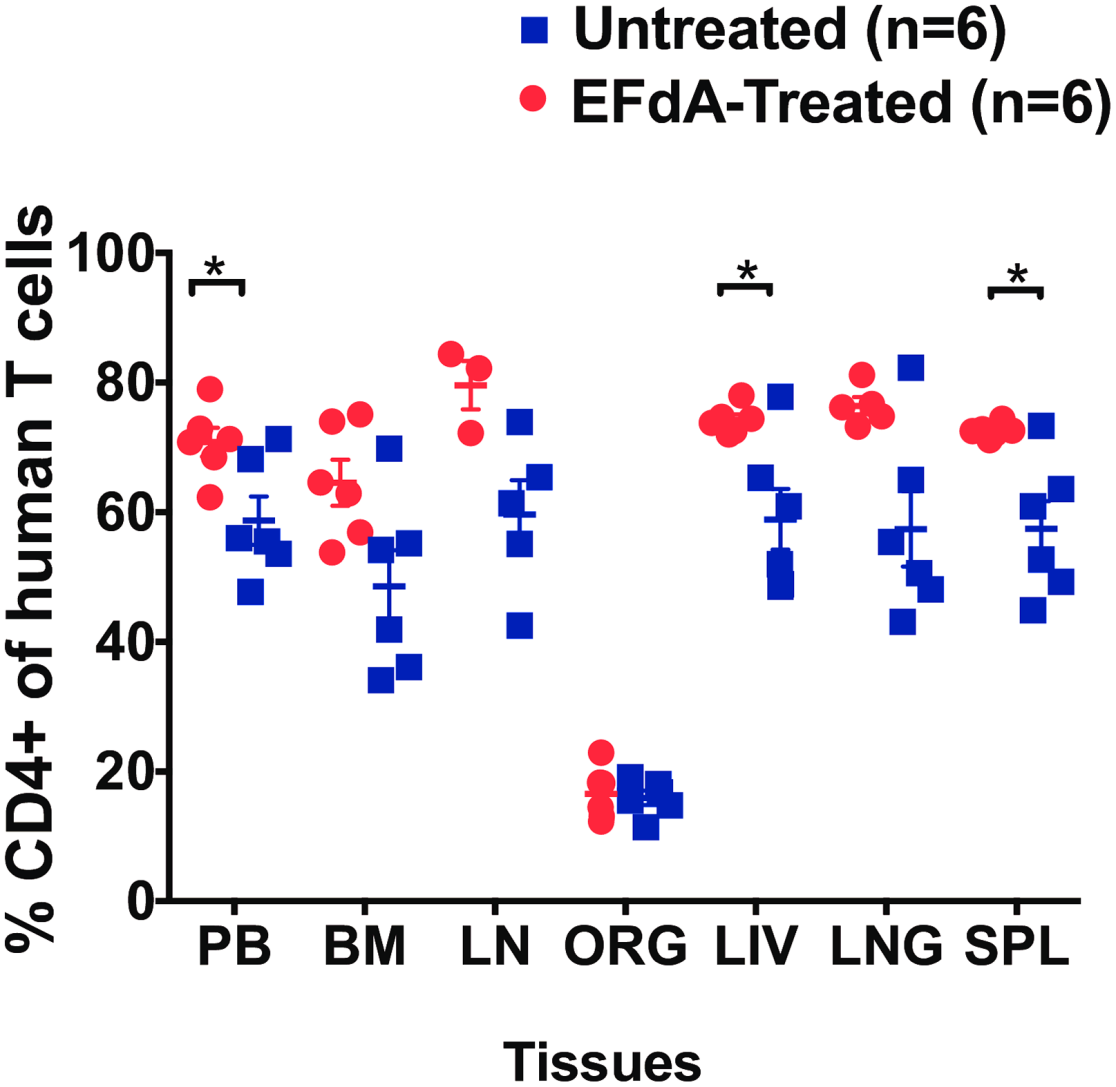
Effect of EFdA treatment on CD4+ T cell levels in the PB, lymphoid and effector tissues of HIV-infected BLT Mice. Percentage of CD4+ human T cells were measured in the peripheral blood (PB), bone marrow (BM), lymph node (LN), organoid (ORG), liver, lung and spleen of EFdA-treated (n = 6, red circles) and untreated (n = 6, blue squares) BLT mice. Horizontal and vertical lines indicate the mean and standard error. A Mann-Whitney test was used to compare mean CD4+ T cell level between EFdA-treated and untreated mice (*p<0.05).

Suppression of plasma viremia below detection levels is not often associated with a significant increase in CD4+ T-cell counts [29]. In this study, we measured CD4+ T cell levels in the PB of BLT mice prior to HIV infection (week 0), 2 weeks post-HIV infection and during EFdA treatment (3–6 weeks). Three weeks post-HIV infection, CD4+ T cell levels were significantly decreased in the PB compared to pre-infection levels (mean: %CD4+ T cells weeks 0 vs 3: 76.2% vs 65.5%; p = 0.001). After 3 weeks of treatment, CD4+ T cell levels in the PB of EFdA-treated mice was significantly higher compared to untreated mice (p = 0.04, Fig 4).

In addition to PB, we also analyzed CD4+ T cell levels in the primary and secondary lymphoid tissues and effector tissues after EFdA treatment. We observed significantly higher levels of CD4+ T cells in the liver (p = 0.04), spleen (p = 0.002) of EFdA-treated mice compared to untreated mice (Fig 4). The CD4+ T cell levels were also higher in the BM, LN, lung of EFdA-treated mice compared to untreated mice, however the difference did not reach significance.

### Oral administration of EFdA results in a strong reduction in HIV-RNA levels in CVL and the FRT

**Fig 5:**
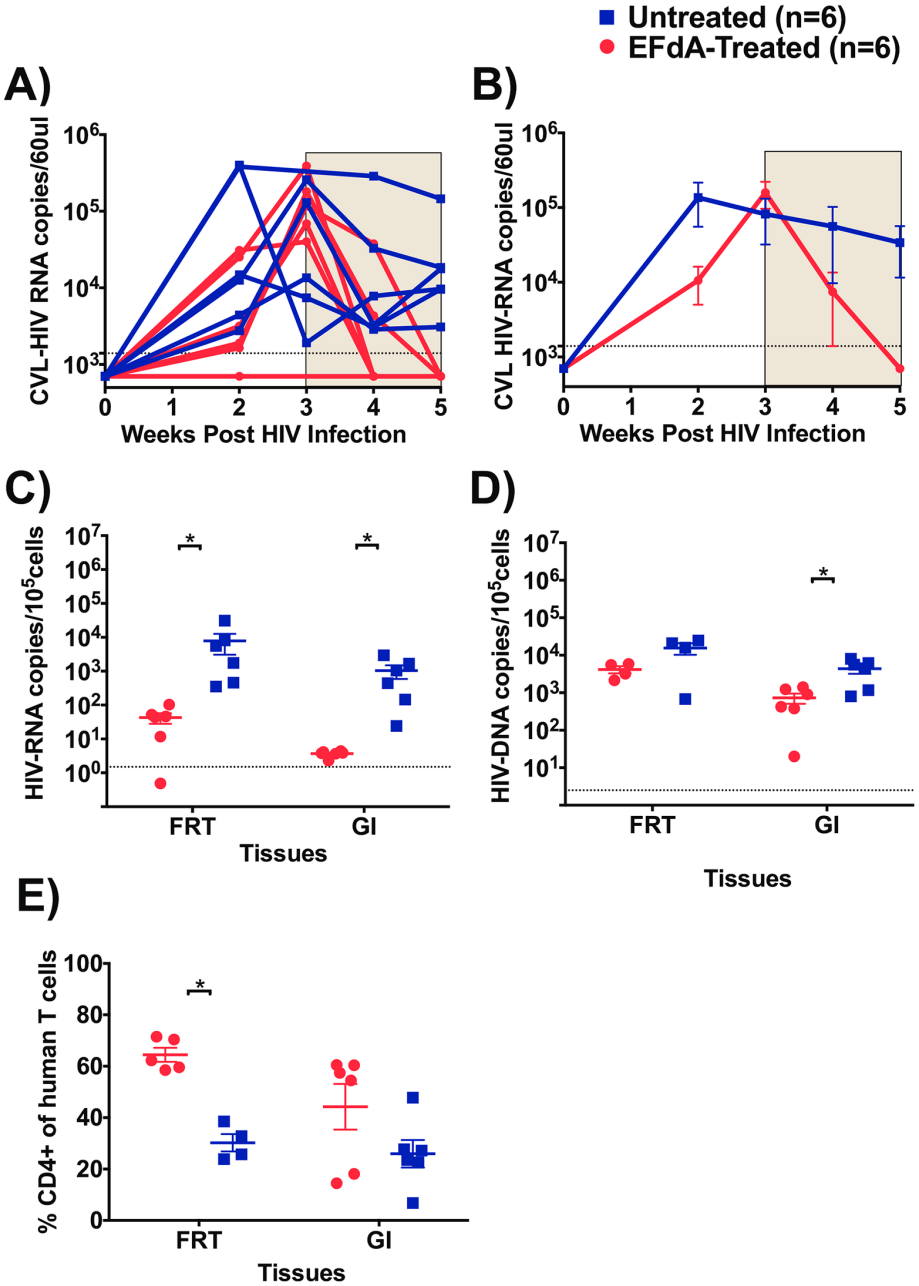
Effect of EFdA administration on HIV-RNA, HIV-DNA and CD4+ T cell levels in FRT and GI tract. A) HIV-RNA levels in cervicovaginal lavage (CVL) of EFdA-treated (n = 6, red lines) and untreated mice (n = 6, blue lines). HIV-RNA was not measured in one EFdA-treated mice at 3^rd^ week B) Mean HIV-RNA levels in CVL of EFdA-treated and untreated mice from panel A. Shaded area indicates the weeks of EFdA (treated) and PBS (untreated controls) administration. C) HIV-RNA, D) HIV-DNA, and E) Percentage of CD4+ human T cell levels in the gastrointestinal (GI) and female reproductive tract (FRT), of EFdA-treated (n = 6, circles, red) and untreated (n = 6, square, blue) HIV-infected BLT mice. Dotted lines indicate the limit of detection (CVL RNA -1400 copiesl/60μl, cell associated HIV-RNA: 1.5 copies/10^5^ cells, HIV-DNA: 2.5 copies/10^5^ cells). Horizontal and vertical lines indicate mean and standard error. Repeated measures of two-way ANOVA was used to compare the mean levels of HIV-RNA in CVL between EFdA-treated and untreated mice. A Mann-Whitney test was used to compare mean HIV-RNA and HIV-DNA levels and CD4+ T cell levels between EFdA-treated and untreated BLT mice (*p<0.05).

In order to establish the effect of EFdA administration on HIV replication in CVL, HIV-RNA levels in CVL were measured prior to treatment initiation and longitudinally during EFdA treatment (Fig 5A and 5B). At the time of treatment initiation, CVL HIV-RNA levels were similar (p = 0.66) between mice in the treatment (mean: 1.5x10^5^ copies/60μl, range: 0.4–3.8x10^5^ copies/60μl) and control groups (mean: 0.8x10^5^ copies/60μl, range: 0.01–2.5x10^5^ copies/60μl). Within 2 weeks of EFdA treatment, CVL HIV-RNA levels were reduced to undetectable levels compared to untreated mice (mean 0.3x10^5^ copies/60μl, range: 0.03–1.4x10^5^ copies/60μl, Fig 5A and 5B). We also measured the levels of HIV-RNA in cells isolated from the FRT after 3 weeks of treatment and we observed more than a 2-log difference (p = 0.002, Fig 5C) in cell-associated HIV-RNA levels in EFdA-treated mice (mean: 42 copies/10^5^ cells, range: 0.4–102 copies/10^5^ cells) compared to untreated mice (mean: 7849 copies/10^5^ cells, range: 351–30927 copies/10^5^ cells). HIV-DNA levels were also lower in the FRT of EFdA-treated mice compared to untreated mice however, this difference did not reach statistical significance (Fig 5D). These findings indicate that EFdA can penetrate into the FRT and efficiently control viral replication.

### EFdA efficiently inhibits HIV replication in the GI tract

The GI tract is an important site of HIV transmission and CD4+ T cell depletion particularly early after infection [30, 31]. In order to investigate the effect of EFdA on viral replication, mononuclear cells were isolated from GI tract 3 weeks after EFdA treatment as described previously [23] and cell-associated HIV-RNA and HIV-DNA levels were measured in EFdA-treated and untreated mice. Over a 2-log difference (p = 0.0002) was noted in the levels of HIV-RNA in the GI tract of EFdA-treated mice (mean: 3.7 copies/10^5^ cells, range: 2.3–4.3 copies/10^5^ cells, Fig 5C) compared to untreated mice (mean: 1042 copies/10^5^ cells, range: 24–2933 copies/10^5^ cells). EFdA treatment also resulted in significantly lower levels of HIV-DNA in the GI tract of EFdA-treated mice (p = 0.04, mean: 728 copies/10^5^ cells, range: 20–1412 copies/10^5^ cells) compared to untreated mice (mean: 4361 copies/10^5^ cells, range: 801–7992 copies/10^5^ cells) (Fig 5D). Together, these results demonstrate the strong ability of EFdA to control HIV replication in the GI tract.

### Effects of EFdA treatment on CD4+ T cell levels in the gastrointestinal and female reproductive tract

Studies have shown a more pronounced depletion of CD4+ T cells in the GI tract mucosa than in PB. Examining the restoration and/or maintenance of CD4+ T cells in GI tract provides a more accurate assessment of the efficacy of ART [32]. Similar to the GI tract, tissues of the FRT contain partially activated, memory CD4+ T cells that can serve as targets for HIV [33] and experimental infection of rhesus macaques with SIV has shown a rapid depletion of CD4+ T cells [34]. In this study, we measured CD4+ T cell levels in CVL of BLT mice prior to HIV infection (week 0), 3 weeks post-HIV infection and during EFdA treatment (3–6 weeks). Three weeks post HIV infection, CD4+ T cell levels were significantly decreased in the CVL of BLT mice (mean: %CD4+ T cells, weeks 0 vs 3: 60.1% vs 5.3%; p = 0.004). Two weeks of EFdA treatment did not result in significant differences in the levels of CVL CD4+ T cells between EFdA-treated and untreated mice (mean: %CD4+ T cells, EFdA-treated mice: 44.3%; untreated mice: 24%, p = 0.12). However, analysis of FRT tissue after 3 weeks of EFdA treatment showed significantly higher levels of CD4+ T cells in the EFdA-treated mice compared to untreated mice (p = 0.01, Fig 5E). The CD4+ T cell levels were also higher in the GI tract of EFdA-treated mice compared to untreated mice, however the difference did not reach significance (Fig 5E).

## Discussion

Efficient treatment of HIV infection with ART can reduce plasma HIV-RNA levels in patients to below the detection limit of clinical assays (50 copies of HIV-RNA/ml). However, some antiretroviral drugs like AZT and lamivudine that are widely used in developing countries have been shown to have a sub-optimal therapeutic response in the FRT and lymphoid tissues leading to persistent HIV replication [2, 27, 28]. This can result in the emergence and transmission of HIV drug resistant variants and chronic inflammation [4, 5]. Therefore, it is imperative to examine the ability of novel HIV treatment strategies to inhibit HIV replication at these sites. In the present study, we used humanized BLT mice to evaluate the systemic antiviral efficacy of EFdA, a novel NRTI, with particular emphasis on the FRT and the GI tract, major sites of HIV transmission and CD4+ T cell depletion [30, 31, 35].

Daily oral EFdA treatment of HIV_JR-CSF_ infected BLT mice for 3 weeks resulted in a decline in plasma HIV-RNA to undetectable levels. Greatly expanding on previous reports [10, 11], here we demonstrate a strong decline in HIV-RNA in CVL to levels below our limit of detection (1400 copies/60μl) showing that EFdA can penetrate into and efficiently reduce virus replication in the FRT. Recently, EFdA has been shown to suppress SIV replication in prostate, seminal vesicles, and lymphoid tissues of one SIV-infected macaque treated for 4 months [9]. To better characterize the ability of EFdA to suppress HIV replication, we performed comprehensive analyses of the levels of HIV-RNA and HIV-DNA in multiple tissues including the FRT and GI tract of EFdA-treated mice. We observed significantly lower levels of cell-associated HIV-RNA in the FRT and GI tract of EFdA-treated mice compared to untreated mice. Furthermore, HIV-DNA levels were lower in the GI tract of EFdA-treated mice compared to untreated mice. Taken together, our results demonstrate that EFdA efficiently penetrates into the FRT and GI tract, the major sites of viral replication. These results strongly suggest that EFdA treatment may also reduce the likelihood of HIV transmission.

There is evidence that the liver, lung, spleen and LN can also serve as possible viral sanctuaries that could further complicate HIV treatment [2, 36]. Hence, we measured HIV-RNA and HIV-DNA levels in primary and secondary lymphoid tissues and effector sites like the liver and lung after 3 weeks of EFdA therapy. We observed significantly lower levels of cell-associated HIV-RNA in primary and secondary lymphoid tissues of EFdA-treated mice compared to untreated mice. In particular, we observed a 2–3 log difference in cell-associated HIV-RNA in the spleen and LN of EFdA-treated mice compared to control mice. In addition, HIV-DNA levels were significantly lower in the BM, LN, ORG, liver and lung. Overall, these findings indicate that EFdA can effectively suppress systemic virus replication including “viral sanctuary” sites like the LN, GI tract and FRT.

Successful clinical outcomes are dependent on adequate recovery of CD4+ T cells following ART [37]. There is now increasing evidence to show that patients failing to achieve CD4+ T-cell counts >500 cells/μl are at increased risk of developing serious non-AIDS events [38], including cardiovascular disease, hypertension, liver disease, non-AIDS malignancies and neurocognitive impairment [39]. In this study, we measured the efficacy of EFdA treatment to prevent depletion of CD4+ T cells in the PB, CVL and tissues including the FRT, GI tract and lymphoid tissues. CD4+ T cell levels in CVL dramatically decreased following HIV infection. After only two weeks of EFdA treatment, we observed a trend towards higher CD4+ T cells in CVL of EFdA-treated mice. These results are consistent with prior studies describing the rapid decline of CD4+ T cells in CVL and FRT following HIV exposure in comparison to the more moderate decline in CD4+ T cell levels in PB. Furthermore, these results are consistent with previous reports describing the gradual restoration of CD4+ T cells in CVL following the initiation of ART [40]. We observed higher levels of CD4+ T cells in the PB and most importantly in the GI tract and FRT tissues of EFdA-treated mice after 3 weeks of treatment compared to untreated mice. Thus, in addition to demonstrating the effectiveness of EFdA in reducing HIV replication, our study also demonstrates the potential clinical benefit of novel EFdA in CD4+ T cell recovery.

In summary, EFdA treatment of HIV-infected BLT mice resulted in a significant reduction in HIV-RNA levels in the PB and CVL to undetectable levels. Importantly, EFdA treatment resulted in a strong reduction in HIV-RNA levels in both the FRT and GI tract tissues. Our results show that EFdA is efficient in penetrating into these tissues and effectively suppresses viral replication. Furthermore, EFdA treatment prevented depletion of CD4+ T cells in the lymphoid and mucosal tissues demonstrating further potential clinical benefits. These pre-clinical studies strongly suggest that EFdA has outstanding characteristics to serve as an excellent component of future ART formulations for HIV therapy and prevention.
